# Identifying Therapeutic Targets for Sepsis Research: A Characterization Study of the Inflammatory Players in the Cecal Ligation and Puncture Model

**DOI:** 10.1155/2018/5130463

**Published:** 2018-08-05

**Authors:** Sara Nullens, Joris De Man, Chris Bridts, Didier Ebo, Sven Francque, Benedicte De Winter

**Affiliations:** ^1^Laboratory of Experimental Medicine and Pediatrics, Division of Gastroenterology, University of Antwerp, Antwerp, Belgium; ^2^Immunology-Allergology-Rheumatology Department, University of Antwerp and Antwerp University Hospital, Antwerp, Belgium; ^3^Department of Gastroenterology and Hepatology, Antwerp University Hospital, Edegem, Belgium

## Abstract

During sepsis, disturbed gastrointestinal motility and increased mucosal permeability can aggravate sepsis due to the increased risk of bacterial translocation. To help identify new therapeutic targets, there is a need for animal models that mimic the immunological changes in the gastrointestinal tract as observed during human sepsis. We therefore characterized in detail the gastrointestinal neuroimmune environment in the cecal ligation and puncture (CLP) model, which is the gold standard animal model of microbial sepsis. Mice were sacrificed at day 2 and day 7, during which gastrointestinal motility was assessed and cytokines were measured in the serum and the colon. In the spleen, lymph nodes, ileum, and colon, subsets of leukocyte populations were identified by flow cytometry. Septic animals displayed an impaired gastrointestinal motility at day 2 and day 7. Two days post-CLP, increased serum and colonic levels of proinflammatory cytokines were measured. Flow cytometry revealed an influx of neutrophils in the colon and ileum, increased numbers of macrophages in the spleen and mesenteric lymph nodes, and an enhanced number of mast cells in all tissues. At day 7 post-CLP, lymphocyte depletion was observed in all tissues coinciding with increased IL-10 and TGF-*β* levels, as well as increased colonic levels of IL-17A and IFN-*γ*. Thus, CLP-induced sepsis in mice results in simultaneous activation of pro- and anti-inflammatory players at day 2 and day 7 in different tissues, mimicking human sepsis.

## 1. Introduction

Ileus, defined as the inhibition of the propulsive gastrointestinal (GI) motility of the entire GI tract, is a common complication often observed following surgical manipulation (so-called postoperative ileus or “POI”) of the GI tract or during sepsis. Sepsis on the other hand remains a leading cause of mortality in intensive care units worldwide [1, 2]. Ileus together with the occurrence of mucosal barrier dysfunction not only results from sepsis but will contribute to and sustain it by the translocation of bacteria and (bacterial) antigens [3–5]. Neuronal cells as well as inflammation play a pivotal role in the initiation and maintenance of ileus [6–8]. On the one hand, immune cells are able to secrete mediators that directly affect smooth muscle cells or activate neuronal reflex pathways, resulting in motility disturbances, pain, and/or hypersensitivity. Activation of mechanisms of immune homeostasis may thus result in inflammation [9]. On the other hand, neurotransmitters secreted from neurons affect immune cells; this feature has been demonstrated for mast cells, T cells, and dendritic cells [10–14]. The pathophysiology of POI and sepsis-induced ileus has been extensively studied [6–8]. The role of the aforementioned mast cells and macrophages has been unraveled [15–17], but much remains to be elucidated on the role of other leukocytes, such as T cells, in the GI tract during sepsis-induced ileus. Inflammation and leukocyte recruitment play a major role during endotoxemia-induced ileus [18], a feature that is also present in POI [19]. Both disease states thus share many characteristics.

Results obtained from animal research in sepsis are often difficult to extrapolate towards the human clinical setting [20–22]. The cecal ligation and puncture (CLP) model, by many considered to be the gold standard animal model with regard to sepsis research, adequately mimics the hemodynamic and metabolic features of the human septic patient [23]. Data on the development of CLP-induced GI inflammation and motility disturbances are, however, limited. Overhaus et al. elegantly showed that performing the CLP procedure in rats resulted in an impaired GI motility, associated with an influx of neutrophils and monocytes and subsequent increased levels of inducible nitric oxide synthase (iNOS), interleukin (IL)-6, IL-1*β*, and monocyte chemoattractant protein-1 (MCP-1) in the colonic muscle layers [17]. Most research was limited to the study of the muscularis externa, as it harbours a dense network of immune cells in direct interplay with smooth muscle cells [24]. The lamina propria however constitutes a major locus of antigen presentation in the GI tract [25, 26] and should therefore not be overlooked in the elucidation of the pathogenesis of sepsis-induced ileus.

In human sepsis, the majority of data obtained on the role of leukocyte subsets in sepsis is confined to the peripheral blood, focusing primarily on the role of the immune-suppressing CD4^+^CD25^+^Foxp3^+^ regulatory T cells (Tregs), impaired monocyte HLA-DR expression, and lymphocyte apoptosis [27–29]. In contrast, much remains to be elucidated on the role of different leukocyte subsets in specific tissues and organs during sepsis.

Detailed study of the neuroimmune GI environment during sepsis could point researchers towards new therapeutic perspectives that tackle the impaired mucosal barrier function and/or disturbed GI motility. We therefore aimed at thoroughly characterizing the gastrointestinal neuroimmune environment in the cecal ligation and puncture model in time. Gastrointestinal motility as well as inflammation, systemically in the blood as well as locally in the colon, was measured. Flow cytometry was performed on the spleen, mesenteric lymph nodes (MLN), ileum, and colon to further characterize the immune cell subsets that could be of importance in the development of sepsis-induced ileus. We specifically aimed at identifying a myriad of immune cells within a single tissue sample in a specific time frame following CLP-induced sepsis, focusing on the different types of T cells (CD4^+^ helper T cell, CD8^+^ cytotoxic T cell, and Tregs), as well as on the different subsets of DCs in the gut.

Finally, by adjusting the CLP procedure, we aimed to obtain an animal model that exhibits the different immune phases that have been typically described in human sepsis, namely, the systemic inflammatory response syndrome (SIRS) and the compensatory anti-inflammatory response syndrome (CARS) or anergic phase [30].

## 2. Materials and Methods

### 2.1. Animals

Eight-week-old male Swiss OF-1 mice were obtained from Charles River (France) and were allowed to acclimatize for two weeks in their cages (6 mice per cage) under specific conditions (12 h light-dark cycle, 21 ± 1°C, 40–60% humidity) with unlimited access to regular chow and tap water. All experiments listed below were approved by the Ethical Committee on Animal Experiments by the University of Antwerp (file number 2012-42 and 2012-42-*extension*).

### 2.2. Cecal Ligation and Puncture

Sepsis was induced by means of a CLP procedure as previously described by us and others [23, 31, 32]. In short, OF-1 mice were anesthetized using a mixture of ketamine (60 mg/kg intraperitoneally (i.p.)) and xylazine (6.67 mg/kg i.p.) and placed on a heating pad in the supine position. A midline laparotomy was performed after abdominal shaving and disinfection with a polyvidon-iodine solution. The cecum was exteriorized and positioned onto moist sterile cottons, and cecal contents were gently pushed towards the distal cecum. Different approaches to the CLP model were studied in order to obtain a reproducible animal model without notable mortality in order to minimize the number of animals lost to the procedure: the cecum was either ligated at 50 or 75% of its length with a 4/0 silk thread and subsequently punctured once or twice through-and-through with a 21, 23, or 25G needle. The cecum was subsequently gently manipulated as to protrude a small but consistent amount of stool from the puncture holes. The ligated cecum was repositioned into the abdominal cavity, and the abdomen was closed in layers with 5/0 ETHILON sutures. Mice received 1 ml of 37°C saline s.c. for fluid resuscitation and 0.05 mg/kg of buprenorphine subcutaneously (s.c.) for pain relief. Mice were allowed to recover in a heated cage (28°C) with free access to water. Sham-operated mice received a midline incision without ligation or puncturing of the cecum.

### 2.3. Survival Analysis and Clinical Disease Score

Mice were monitored until 14 days following the CLP or sham procedure. Animals were weighed twice daily (8 a.m. and 7 p.m.) and assessed individually for signs of illness by means of a modified clinical disease score (CDS) (Supplementary Table 1) [33, 34], resulting in a CDS ranging between 0 (no signs of illness) and 15 (maximum). Mice were prematurely sacrificed when they lost over 15% of their baseline body weight, when they appeared moribund or had a CDS > 8. Data from animals that were prematurely sacrificed or had succumbed prior to the experiments were only included in the survival analysis.

### 2.4. Experimental Design

Based upon the aforementioned survival analysis and clinical parameters (vide infra Survival Analysis of the Implemented Model), two time points were included for the following analyses. Animals were studied either 48 hours (CLPd2) or 7 days (CLPd7) following the procedure. Control animals were studied 2 days following the procedure (sham).

In a first set of experiments, GI transit was assessed by means of the solid beads method (*n* = 8–10/group) in order to ascertain the occurrence of ileus, whereupon animals were anesthetized and sacrificed by means of cardiac puncture. Whole blood samples were utilized to obtain a cell blood count and white blood cell differential on the one hand and to obtain whole blood cultures on the other hand. The proximal colon was harvested for cytokine analysis by means of RT-PCR and cytometric bead array (CBA), as well as histology and immunohistochemistry. Finally, mesenteric lymph nodes were harvested for cultures as well.

In a second set of experiments, serum samples were obtained by cardiac puncture for cytokine analysis. Spleens and draining MLN were harvested for flow cytometric analysis (*n* = 10–12 in each group). Furthermore, lamina propria mononuclear cells (LPMCs) were isolated from the ileum and colon at the same time for the remainder of the flow cytometry experiments.

### 2.5. In Vivo Measurement of Gastrointestinal Transit: The Solid Beads Method

Mice were overnight deprived of food with unlimited access to tap water. Mice were given an oral gavage with 0.5 ml of tap water containing 25 glass green-colored beads (diameter 0.3 mm) through a 20G flexible catheter (Terumo; outer diameter 1.10 mm, inner diameter 0.80 mm). Mice were sacrificed 2 h following the gavage, and the GI tract was resected and divided into 10 parts (stomach, 5 small bowel segments, cecum, proximal colon, distal colon, and faeces). The number of beads in every segment was counted under a stereomicroscope for calculation of percentage gastric emptying (% GE) and the geometric center of intestinal transit (GC) as a marker for overall GI transit [35].

### 2.6. Peripheral Blood Count

Following anesthesia, animals were sacrificed 48 h or 7 days following the CLP or sham procedure with cardiac puncture while obtaining EDTA-treated blood samples. A cell blood count and white blood cell differential were obtained with the Advia®120 Haematology Analyzer using the Perox method.

### 2.7. Cytokine Measurements

Blood samples (Multivette® 600 capillary blood collection, Sarstedt) were centrifuged (5000 rpm, 5 min, 20°C) and supernatants were stored at −80°C until further analysis by means of CBA (BD) according to the manufacturer's instructions.

Colonic cytokine levels were determined at the protein level as well as the mRNA level. For the levels of secreted protein, whole colons were rinsed with phosphate buffered saline, blotted dry, weighed and placed in RPMI medium supplemented with 2 mM glutamine, 100 U/ml penicillin, 100 *μ*g/ml streptomycin, and 10% fetal bovine serum (“full RPMI”), and incubated for 24 h (37°C, 95% O_2_, 5% CO_2_). Supernatants were collected 24 h later and assessed for levels of IL-6, TNF-*α*, IL-2, IL-17A, IFN-*γ*, and IL-10 (pg/g colon) using the BD CBA Mouse Cytokine Kit.

To determine the cytokine content at the mRNA level, total RNA was isolated from a snap-frozen piece of proximal colonic tissue using the Qiagen RNeasy Mini Kit. Total RNA was treated with DNase and converted to cDNA using the Transcriptor First Strand cDNA Synthesis Kit (Roche Applied Science). Quantitative real-time PCR was performed using the TaqMan® Universal PCR Master Mix (Life Technologies). The following primers were used: IL-6 (gene id: 16193-Mm00446190_m1), TNF-*α* (gene id: 21926-Mm00443258_m1), IL-10 (gene id: 16153-Mm00439614_m1), IL-17A (gene id: 16171-Mm00439618_m1), IFN-*γ* (gene id: 15978-Mm01168134_m1), IL-1 alpha (gene id: 16175-Mm00439620_m1), IL-1 beta (gene id: 16176-Mm00434228_m1), CRP (gene id: 12944-Mm00432680_g1), and TLR4 (gene id: 21898-Mm00445273_m1). The PCR reaction was performed in a 25 *μ*l reaction volume with the following amplification parameters: 50°C for 2 min, 95°C for 10 min, followed by 40 cycles of 95°C for 15 sec, and 60°C for 1 min. Out of the three housekeeping genes included in the PCR reaction (GAPDH, beta-actin, and eEF-2), GAPDH was determined to be the optimal housekeeping gene to which the expression of genes of interest was normalized [36–39].

### 2.8. Cultures of Blood and Mesenteric Lymph Nodes

One drop of EDTA-treated full blood was obtained by cardiac puncture from the animals in which colonic permeability was studied and plated onto a blood agar culture plate following enrichment and incubated at 37°C for 24 h in ambient air supplied with 5% CO_2_. Additionally, mesenteric lymph nodes (MLN) were resected aseptically, suspended in RPMI 1640 medium (Gibco, LifeTechnologies), and mashed manually using a 10 ml syringe plunger through a 40 *μ*m nylon cell strainer. Homogenized MLN were plated onto a blood agar culture following enrichment.

### 2.9. Flow Cytometric Characterization of Gastrointestinal Tissues

#### 2.9.1. Preparation of Single Cell Suspension from the Spleen and Mesenteric Lymph Nodes

Flow cytometric identification of different immune cells was performed as described elsewhere [37, 40, 41]. In short, spleens and MLN collected in full RPMI medium were manually dissociated applying a 10 ml syringe plunger over a 40 *μ*M cell strainer (Falcon®, BD) and rinsed with RPMI followed by 5 min centrifugation at 4°C, 1500 rpm. Cell pellets were subsequently incubated with 5 ml red blood cell lysis buffer (Sigma-Aldrich) followed by washing and suspension in RPMI and storage on ice until further processing of samples. Cells were counted and viability was assessed using the Trypan blue staining method.

#### 2.9.2. Isolation of Lamina Propria Mononuclear Cells from the Ileum and Colon

Colonic and ileal LPMCs were isolated as described previously [25, 26, 42–44]. In short, GI tissues were opened longitudinally following meticulous removal of mesenterium and Peyer's patches and rinsed of GI contents with calcium- and magnesium-free Hank's balanced salt solution (HBSS). Tissues were cut into 5 mm pieces and incubated in an Erlenmeyer flask for 20 min in HBSS supplemented with 1 mM EDTA and 2 mM dithiothreitol at 37°C on a stir plate at 220 rpm, followed by thorough washing with HBSS. This was repeated 4 to 6 times depending on the turbidity of the supernatants. Tissues were then incubated in “lymphocyte growth medium” (LGM) (full RPMI supplemented with 25 mM HEPES buffer, 1 mM sodium pyruvate, 1% MEM nonessential amino acids 100x, 2% MEM essential amino acids 50x, and 50 *μ*M *β*-mercaptoethanol) containing 1 mg/ml collagenase and 5 m mM CaCl_2_ for enzymatic digestion. Tissues were subsequently further dissociated mechanically using the plunger of a 1 ml syringe. To remove debris, the suspension was sieved through wet gauze layered in a funnel. The cell suspension was then washed with LGM (10 min, 1500 rpm) and layered onto a 30 : 70% Percoll gradient followed by centrifugation for 20 min at 3500 rpm at room temperature. Cells were harvested at the Percoll interface, washed twice, resuspended in 2 ml LGM, and stored on ice until further processing. Cells were counted and viability was assessed using the Trypan blue staining method.

#### 2.9.3. Flow Cytometry Analysis

**Figure 1:**
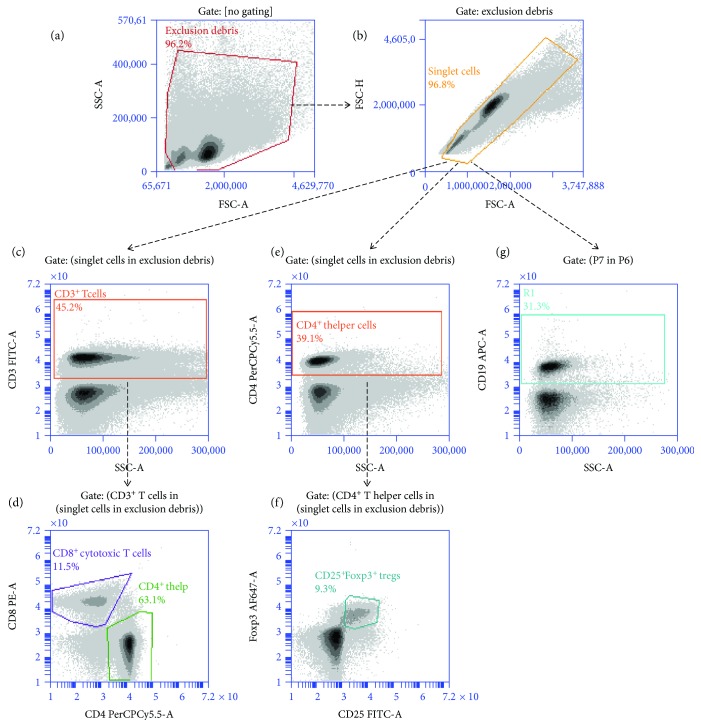
Example of a gating strategy for flow cytometry (panel 3—T cell and B cell stain and panel 4—Treg stain). Leukocytes were gated based upon their forward scatter (FSC) and side scatter (SSC) properties in order to exclude debris (a), followed by the exclusion of doublets based upon the forward scatter height (FSC-H) versus the forward scatter area (FSC-A) plot (b). Next, the percentage of CD3^+^ cells (effector T cells) was identified in the singlet cells gate gated upon the expression of CD3 and SSC properties (c). Subsequently, the differentiation was made in this population between the number of CD4^+^ cells to identify helper T cells or CD8^+^ cells to identify cytotoxic T cells (d). In the singlet gate, the percentage of CD4^+^ cells (helper T cells) was then identified (panel 2) (f) and subsequently the CD25^+^Foxp3^+^ cells were identified in the CD4^+^ gate (f) to identify regulatory T cells. In the leukocyte gate, the percentage of CD19^+^ cells was also determined to identify B cells (g). Cell subsets were expressed as a percentage of the total leukocyte count. FMO samples were implemented as appropriate gating controls. A: area; CD: cluster of differentiation; Foxp3: forkhead box p3; FSC: forward scatter; H: height; SSC: side scatter; Treg: regulatory T cells.

Flow cytometric analysis was performed using the BD Accuri™ C6 flow cytometer (Becton Dickinson, Erembodegem, Belgium (instrument specifics—Supplementary Table 2)). Five staining panels were designed in order to stain a substantial number of immune cells (Table 1). In short, 1 × 10^6^ cells were incubated for 10 min at 4°C with 50 *μ*l rat serum (1/50) to evade nonspecific staining. Next, cells were washed and stained with 100 *μ*l staining mix for 40 min at 4°C in the dark. All antibodies were diluted 1/100, with the exception of anti-mouse F4/80 APC (1/200) (for a comprehensive list of the antibodies used, see Supplementary Table 3). “Fluorescence minus one” samples (FMO) were included for determining the correct negative populations and gate settings [45–47]. A separate propidium iodide (PI) stain was included to assess for cell viability. Samples were subsequently washed twice, resuspended in 400 *μ*l FACS buffer, and kept on ice in the dark until analysis. Compensation settings were included as appropriate, and the whole sample was measured without a stopping gate (typically between 0.5 × 10^6^ and 1 × 10^6^ events). Results were analyzed using FCS Express 4 software (De Novo Software). As an example, the gating strategy for the T cell and B cell stain is outlined in Figure 1. In short, for all samples, cell aggregates were excluded based upon the forward scatter area versus forward scatter height plot (FSC-A/FSC-H). Then, leukocytes were gated based upon their FSC-H and side scatter H (SSC-H) characteristics in order to exclude debris [40, 42, 48]. Finally, cell populations were defined based upon their expression of several cell surface markers (Supplementary Table 4) and cell subsets were expressed as a percentage of the total leukocyte count in each studied tissue.

### 2.10. Histology and Immunohistochemistry

As macroscopic examination revealed no obvious alterations in colonic segments obtained from septic or control animals (no ulcerations, strictures, or hyperemia whatsoever), we only assessed inflammation of GI tissues at the microscopic level. A full thickness segment (0.5 × 0.5 cm) was taken from the proximal colon immediately adjacent to the cecum. The segment was fixed for 24 h in 4% formaldehyde and subsequently embedded in paraffin. Stainings for lymphocytes (CD3) were performed as follows: paraffin-embedded sections were deparaffinized, endogenous peroxidase was blocked (5 min), and samples were treated with trypsin solution at 37° (10 min) prior to antigen retrieval in a citrate buffer (microwave, pH 6.0). Tissue slices were exposed to anti-CD3 antibody (1 : 300 dilution) or the ApopTag Plus Peroxidase in Situ Apoptosis Kit (Millipore S7101) overnight and subsequently incubated with a biotinylated anti-rabbit secondary antibody. All antibodies were obtained from Abcam.

### 2.11. Statistical Analysis

Data are presented as mean ± SEM, with “*n*” representing the number of mice. One-way ANOVA followed by post hoc Bonferroni analysis or the nonparametric Kruskal–Wallis test was applied when appropriate to compare the results of CDS, rectal temperature, motility parameters, cytokine levels, PCR, and other data. For the survival analysis, the Kaplan–Meier estimator with log-rank test was used. Data from animals that were sacrificed prematurely or had succumbed prior to the experiments were not included in the final analysis. Data were analyzed using SPSS version 20.0 (IBM, Chicago) and visualized using GraphPad Prism version 5.00. Flow cytometry results were processed using FCS Express 4 Flow Research Edition, and CBA data were analyzed using FCAP Array (BD).

## 3. Results

### 3.1. Survival Analysis of the Implemented Model

**Figure 2:**
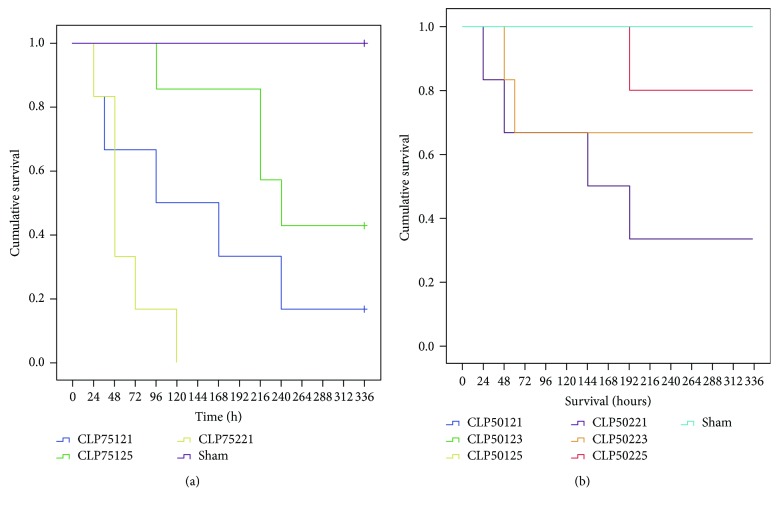
Survival of animals during the CLP procedure. The cumulative survival of animals that underwent the CLP procedure or sham-operated control animals up until 14 days following the procedure. In (a), the cecum was ligated at 75% of its length, whereas in (b), it was ligated at 50% of its length. Note the influence of the needle thickness on survival in (a), with the large bore needles resulting in a higher mortality rate. When ligating 50% of the cecum's length, no mortality was observed when only one puncture was performed independent of the needle size (b). The needle size was then again of influence once two punctures were performed. *n* = 6 animals/group. CLP: cecal ligation and puncture; interpretation of the numeric code used following “CLP”: the first two numbers represent the percentage of the cecum that was ligated, the third number represents how many times the cecum was punctured, and the final two numbers represent the size of the needle, for example, CLP50125 means CLP procedure in which the cecum is ligated at 50% of its length and punctured once (1) with a 25G needle.

Several modifications of the CLP model were studied prior to deciding which procedure would be implemented. In the animals in which 75% of the cecum's length was ligated, a drop in survival could be observed that was furthermore dependent on the needle's size and the number of punctures that were applied (Figure 2(a)). Two punctures with a 21G needle resulted in 100% mortality by day 5, whereas 45% of the animals that had received one puncture with a 25G needle survived until two weeks following the procedure. A milder procedure was subsequently tested, in order to minimize the number of casualties. When 50% of the cecum's length was ligated, all animals survived until two weeks after the procedure but only when receiving a single puncture (no difference between the use of a 21G, 23G, or 25G needle). Once again, mortality increased when animals received two punctures and mortality was highest in association with the large bore needle of 21G (Figure 2(b)). Based on these results, we opted to further characterize the model in which the cecum was ligated at 50% of its length combined with a single 25G puncture, as no mortality in this group was witnessed. Interestingly, CLP50/1/25 animals displayed a biphasic course of their clinical signs of disease, based upon behavior and fluctuations in weight, demonstrating outspoken sickness behavior at day 2 and at day 7 following CLP. We therefore choose to characterize the CLP model in mice at those two time points.

### 3.2. Clinical Disease Score and Gastrointestinal Motility

**Figure 3:**
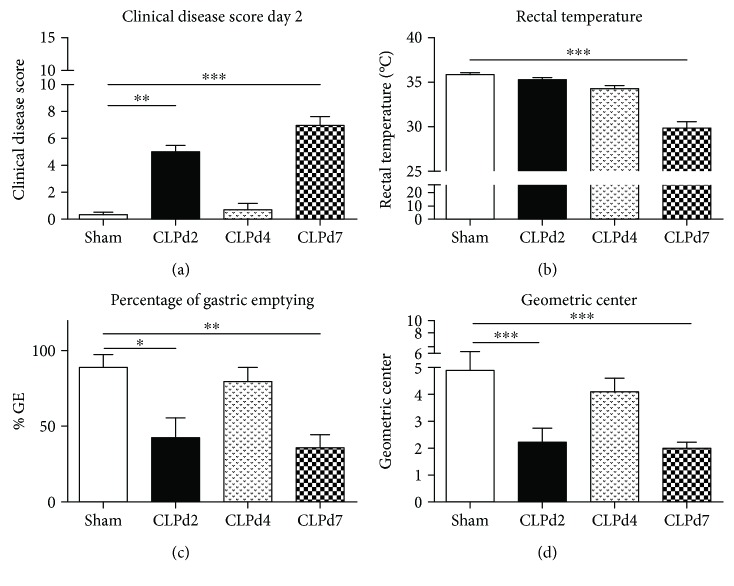
Clinical disease score, temperature, and gastrointestinal motility in the CLP model. Clinical disease score (a), rectal temperature (b), and gastrointestinal motility parameters (percentage of gastric emptying (c) and geometric center (d), as determined by means of the solid beads method) following the CLP50/1/25 procedure at different time points postprocedure (day 2, day 4, and day 7). One-way ANOVA followed by post hoc Dunnett testing (with sham as the control group) or its nonparametric equivalent as appropriate (Kruskall–Wallis test with Dunn's post hoc testing); *n* = 8–10/group, ^∗^ *p* < 0.05, ^∗∗^ *p* < 0.01, ^∗∗∗^ *p* < 0.001.

No control or septic animals allocated to the CLPd2 group succumbed prior to sacrifice, and three animals allocated to the CLPd7 group were sacrificed on the sixth day post-CLP due to moribund appearance. Septic animals displayed a significant increase in their clinical disease score at day 2 as well as at day 7 following the CLP procedure in comparison to sham-operated animals (Figure 3(a)). The rectal temperature was only significantly decreased 7 days post-CLP (Figure 3(b)). GI motility assessment, measured by the solid beads method, demonstrated a significant decrease in %GE as well as the GC both at day 2 and at day 7 following CLP (Figures 3(c) and 3(d)). Four days post-CLP, in between the first and second “sick” phases, no differences were observed concerning signs of disease or GI motility between septic and control animals, clearly underlining the biphasic profile witnessed in this model.

### 3.3. Cell Blood Count

At CLPd2, animals displayed a marked drop in thrombocytes. The total number of leukocytes remained unaltered; however, an obvious lymphocytopenia and monocytosis developed. At day 7 following CLP, animals displayed a profound anemia, thrombocytosis, and leukocytosis with a reduced number of neutrophils and lymphocytes and an increased number of monocytes and immature cells in comparison to the control animals (Table 2).

### 3.4. Cytokine Measurements

Serum cytokine analysis demonstrated the development of a profound proinflammatory state 48 h following CLP, as was demonstrated by a significant rise in the levels of IL-6 and TNF-*α* compared to those of nonseptic control animals. The concentrations decreased towards baseline levels 7 days following the procedure; at this time, serum IL-17A levels were significantly upregulated (Table 3(a)). In line with these results, we observed at the colonic level a tendency towards higher concentrations of IL-17A secreted in the supernatants, without reaching statistical significance. Furthermore, the colonic secreted TNF-*α* and IL-10 concentrations were significantly increased at day 2 and remained significantly upregulated by day 7 (Table 3(b)). mRNa expression of the aforementioned results in the colon corresponded with levels measured in the colonic supernatants, but significance was only reached for TNF-*α* at day 2 and for TNF-*α*, IL-17A, and IFN-*γ* at day 7 (Table 3(c)). Moreover, mRNA expression of C-reactive protein as well as IL-1 beta was significantly upregulated at day 2, confirming the ongoing proinflammatory state. Remarkably, no statistical difference was found in the expression of TLR4, the endotoxin receptor, at both time points.

### 3.5. Cultures of Blood and Mesenteric Lymph Nodes

All mesenteric lymph node cultures from septic animals at day 2 were positive following 24 h of incubation: all displayed the Gram-negative rod *E. coli*, whereas 2 out of 5 displayed the Gram-positive *S. aureus* as well (polymicrobial sepsis). All cultures from control animals remained negative. Blood cultures were all negative in control animals, whereas 3 out of 5 cultures from septic animals again displayed the Gram-negative *E. coli*.

### 3.6. Flow Cytometric Characterization of Gastrointestinal Tissues

#### 3.6.1. T Cell and B Cell Staining

**Figure 4:**
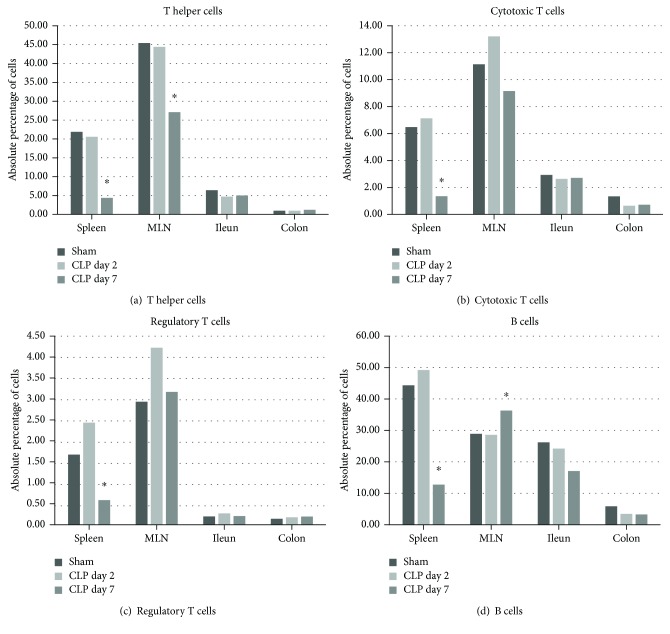
T cell subsets and B cells in different organs during CLP. Schematic representation of the absolute percentage of different T cell subsets and B cells in the spleen, mesenteric lymph nodes, and lamina propria of the ileum and colon. One-way ANOVA with post hoc Dunnett or the Kruskal–Wallis test with post hoc Dunn's test as appropriate with the sham group and the control group. ^∗^ *p* < 0.05 compared to the sham group.

When looking at the specific T cell subset populations, we found that the absolute percentage of T helper cells in the overall leukocyte population dropped significantly in the spleen and MLN at day 7, whereas the absolute percentage of cytotoxic T cells only dropped significantly in the spleen at day 7. We furthermore observed a drop in the absolute number of regulatory T cells at day 7 in the spleen (Supplementary Table 5); however, the relative percentage rose in the MLN and spleen, albeit not significantly in the latter one (relative percentage of Tregs in the overall CD3^+^ T cell subset, for MLN: sham 5.73 ± 0.75%, CLPd2 8.37 ± 0.82%, and CLPd7 9.78 ± 1.34%, *p* = 0.034 for CLPd7 versus sham; for spleen: sham 6.76 ± 1.05%, CLPd2 10.08 ± 1.24%, and CLPd7 9.06 ± 1.59%, *p* = not significant). No obvious differences were observed in the absolute percentage of different T cell subsets in ileal and colonic LPMCs. Concerning the B cell population, we observed a significant drop in the percentages of B cells in the spleen and a trend towards decreased B cell percentages in the ileal LPMC at day 7 post-CLP, whereas their numbers increased significantly in the MLN (Supplementary Table 5). No differences were observed at day 2. A visual overview of these results can be found in Figure 4.

#### 3.6.2. Dendritic Cell Staining

**Figure 5:**
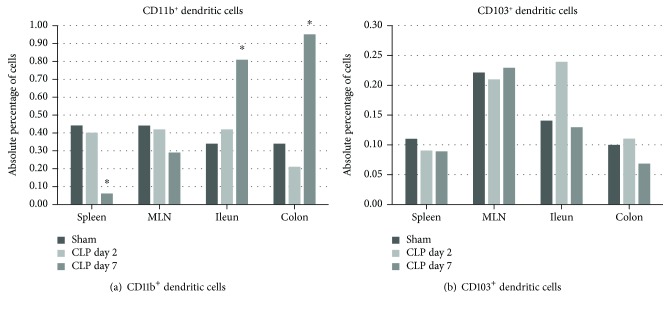
Dendritic cell subsets in different organs during CLP. Schematic representation of the absolute percentage of different dendritic cell subsets in the spleen, mesenteric lymph nodes, and lamina propria of the ileum and colon. One-way ANOVA with post hoc Dunnett or the Kruskall–Wallis test with post hoc Dunn's test as appropriate with the sham group and the control group. ^∗^ *p* < 0.05 compared to the sham group.

When looking at the absolute percentage of CD11b^+^ DCs (MHCII^+^CD11c^+^CD11b^+^) in the overall leukocyte gate, their numbers remained unaffected at day 2 post-CLP in the studied tissue types. At day 7, however, a significant drop in CD11b^+^ DCs could be observed in the spleen, whereas their numbers significantly increased in the colon and ileum (Supplementary Table 5, Figure 5). Concerning the absolute percentage of CD103^+^ DCs (MHCII^+^CD11c^+^CD103^+^) in the overall leukocyte gate, no differences could be observed during sepsis in none of the organs. However, the relative contribution of CD103^+^ DCs to the total number of DCs (%CD103^+^ out of MHCII^+^CD11c^+^) was upregulated in the spleen at day 7 (sham: 9.19 ± 1.36%, CLPd2 9.54 ± 0.87%, and CLPd7 17.03 ± 2.11%, *p* = 0.001 for CLPd7 versus sham) whereas the relative contributions were significantly lowered in ileal (sham: 21.20 ± 3.17%, CLPd2 20.10 ± 1.58, and CLPd7 10.36 ± 1.39, *p* = 0.002 for CLPd7 versus sham) and colonic LPMCs (sham: 16.30 ± 3.00, CLPd2 21.23 ± 5.53, and CLPd7 5.28 ± 0.85, *p* = 0.002 for CLPd7 versus sham).

#### 3.6.3. Other Leukocyte Populations

**Figure 6:**
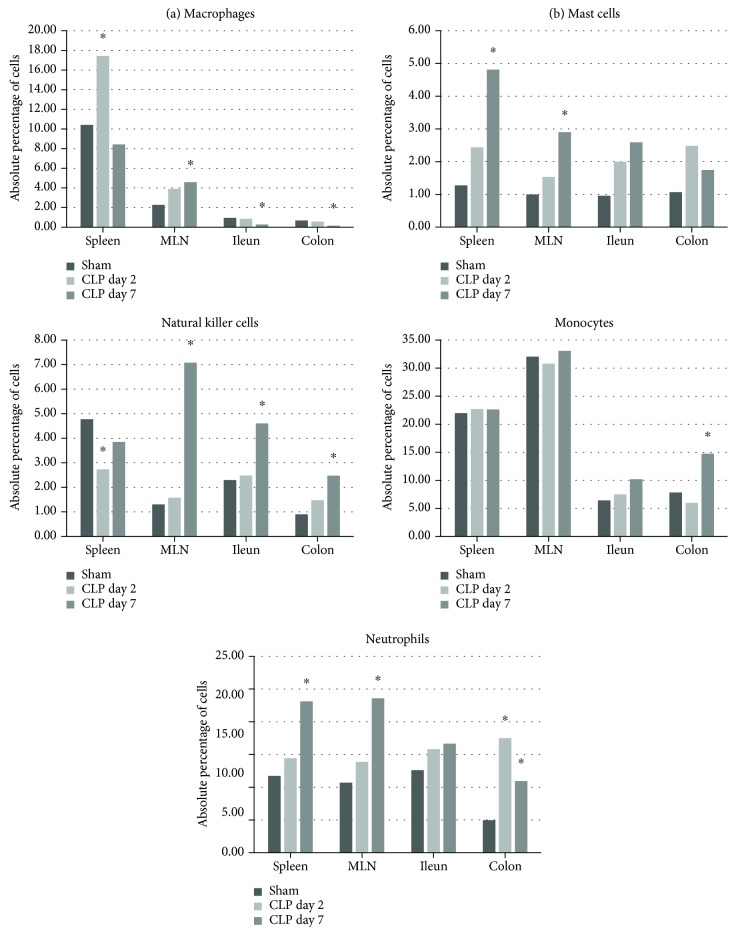
Other leukocyte subsets in different organs during CLP. Schematic representation of the absolute percentage of different immune cell subsets in the spleen, mesenteric lymph nodes, and lamina propria of the ileum and colon. One-way ANOVA with post hoc Dunnett or the Kruskall–Wallis test with post hoc Dunn's test as appropriate with the sham group and the control group. ^∗^ *p* < 0.05 compared to the sham group.

Septic animals displayed a significant increase in the number of F4/80^+^ macrophages in splenic tissue at day 2 and increased numbers in the MLN at day 7 following the CLP procedure. In the ileum and colon, the percentage of macrophages dropped significantly 7 days post-CLP. A significant rise in CD117 (c-Kit) positive cells, presumably mast cells, was observed at day 7 in the spleen and MLN, whereas their numbers remained statistically unaltered in the ileum and colon. When comparing the number of natural killer (NK) cells by quantifying CD335 expression, we observed a significant drop in the percentages in the spleen at day 2, whereas at day 7 post-CLP, the numbers increased significantly in the MLN, ileum, and colon. Ly6C^+^ monocytes remained unaltered in all tissues, with the exception of an increase in their numbers in the colon in animals that suffered from a prolonged sepsis. Ly6G^+^ neutrophils increased significantly in the colon lamina propria at day 2, whereas numbers remained identical in the spleen and MLN. At day 7, however, neutrophil numbers heightened in the spleen and MLN (Figure 6, Supplementary Table 5).

#### 3.6.4. General Summary of the Flow Cytometry Data

Cell viability was >90% in single cell suspensions of the spleen and MLN and between 70 and 80% in the LPMC presumably due to the more aggressive isolation protocol. In short, septic animals displayed no obvious alterations when compared to the sham animals at day 2 in the number of helper T cells in all tissues studied, whereas their numbers dropped significantly in the spleen, MLN, and ileum by day 7 following CLP. The absolute percentage of CD4^+^CD25^+^Foxp3^+^ Tregs in the overall leukocyte population was significantly reduced at day 7 in the spleen; a significant relative increase in the subset of Tregs in comparison to the overall population of T cells was noted in the spleen and MLN. The number of B cells dropped in the spleen, ileum, and colon (whereas in the MLN, an increase in their numbers was observed). This lymphopenia was observed in the blood as well at both time points, compatible with the presence of lymphocyte apoptosis which is a hallmark feature of progressive immunosuppression often observed during sepsis. The numbers of CD8^+^ cytotoxic T cells were only minimally attenuated, and the NK cell subset remained unaffected in the spleen and even increased dramatically in the MLN, colon, and ileum by day 7. CD11b^+^ DCs were significantly higher in the colon and ileum by day 7, whereas their percentages in the spleen and MLN dropped. The relative percentages of CD103^+^ DCs increased in the spleen at day 7 following CLP concomitantly with a drop in the ileum and colon. The percentage of Ly6c^+^ cells (monocytes, but possibly macrophages as well) in the spleen and MLN remained nearly identical to values obtained in control animals, whereas the percentages rose in the ileum and colon, the latter increase being significant. In the blood, a monocytosis was observed from day 2 onwards in septic animals. The opposite was observed concerning the overall percentage of macrophages, as numbers increased significantly at day 2 in the spleen and MLN, whereas numbers in ileums and colons from septic animals remained identical to those in control animals. At day 7, macrophage numbers returned to baseline in the spleen but remained elevated in the MLN. In the colon and ileum, the percentage of F4/80^+^ cells dropped significantly by day 7 post-CLP. At day 2 post-CLP, we quantified a steep rise in the numbers of neutrophils that had infiltrated in the colonic LPMC; by day 7, this increase was also observed in the other tissues, albeit only numerically in the ileum. This rise coincided with the occurrence of neutropenia in the blood of these animals at day 7, which could be suggestive of the migration of neutrophils from the blood towards the inflammatory focus.

### 3.7. Histology and Immunohistochemistry

**Figure 7:**
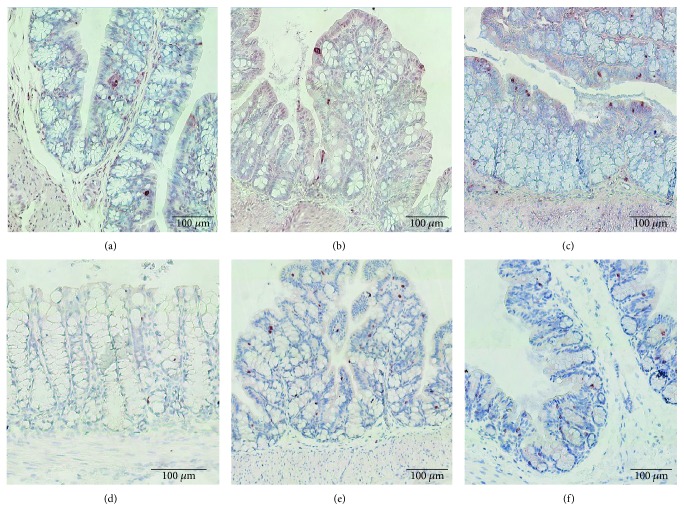
TUNEL and CD3 immunohistochemistry slides. Representative TUNEL staining (a–c) and CD3-staining (d–f) from sham (left), CLPd2 (middle), and CLPd7 (right) animals.

TUNEL staining did not reveal a difference in the number of apoptotic cells between the three groups (sham: 2.13 ± 0.29%, CLPd2 1.75 ± 0.38%, and CLPd7 2.08 ± 0.53, *p* = not significant). The number of CD3 staining T cells was significantly increased at day 2 following CLP in comparison to the control animals and was normalized back again at day 7 (sham: 0.64 ± 0.02, CLPd2 1.31 ± 0.26, and CLPd7 0.86 ± 0.17, *p* = 0.04 for CLPd2 versus sham) (Figure 7).

## 4. Discussion

By performing the cecal ligation and puncture (CLP) procedure, we were able to reproducibly elicit sepsis in mice, as was marked by the occurrence of behavioral characteristics of disease (e.g., impaired mobility and grooming behavior of the animals), weight loss, and the rise in proinflammatory cytokine levels in the serum as well as the colon. All mesenteric lymph node cultures from septic animals at day 2 were positive following 24 h of incubation, whereas cultures from control animals remained negative. In addition, one might determine the levels of LPS in biological samples in order to confirm its secretion by Gram-negative bacteria. The amount of serum samples and supernatants left however in our study was limited, and cross contamination of samples with endotoxin is known to lead to false-positive results; we therefore chose not to perform this analysis in the current setup. Furthermore, the presence of ileus was confirmed 2 days and 7 days after the procedure with a remarkable normalization of the intestinal motility at day 4. Inflammation at day 2 following the CLP was typified by an increase of IL-6 and TNF-*α* in the serum, and a marked rise in TNF-*α* and IL-10 in the colon. At day 7, serum levels of the aforementioned cytokines normalized to those in control animals in the serum but not in the colon. Interestingly, a significant rise was found in the serum concentration of IL-17A and a trend in the colon (*p* = 0.06) at day 7. RT-PCR data could confirm the increased presence of mRNA for IL-17A and TNF-*α*, as well as a significantly upregulated IFN-*γ* mRNA level at day 7 post-CLP. CRP and IL-1 beta mRNA were significantly increased at day 2 in colonic tissue, reflecting the proinflammatory state at the single-organ level as well. No differences however were observed in the TLR4 mRNA levels in colonic tissue in this experiment. Older studies have demonstrated that the upregulation of TLR4 mRNA in vital organs was shown to be associated with a higher mortality [49]. These studies only focused on peripheral blood and spleen samples, and we applied an animal model with no mortality. In a more recent descriptive paper on critically ill colectomized patients, no difference could be found in the TLR4 expression on histological examination of colonic samples from patients and controls [50]. Much therefore remains to be elucidated on the role and dynamic expression of the Toll-like receptors on gastrointestinal tissue during sepsis.

Next, we characterized several types of immune cells in the organs that constitute part of the immune balance between the gut and the environment.

In summary, a profound depletion in the percentages of CD4^+^ as well as CD8^+^ T cells and B cells was observed most evidently at day 7 in the spleen; also, in other tissues, the T cell populations showed a significant decrease, coinciding with an increase in the number of NK cells, albeit the latter not in the spleen. These observations are in line with existing data on human splenic tissue samples from septic patients, where a profound depletion of CD4^+^ T cells and B cells coincided with a relatively stable number of CD8^+^ T cells and NK cells [51, 52]. Moreover, these results confirm the previous flow cytometric observations made by Sharma et al. in T cell subsets isolated from the spleens of mice 20 h post-CLP [53]. CD335, also termed NKp46 or natural cytotoxicity receptor, is however also expressed on the cell surface of innate lymphoid cell type 3 located in the intestinal lamina propria [54, 55]. We therefore cannot exclude that the number of NK cells also includes a certain percentage of type 3 innate lymphoid cells. The above findings in part endorse our hypothesis that the CLP model is an immunologically qualified model to study sepsis, with many immunological features that resemble the human disease state of “sepsis.”

Many research has been conducted on the role of the regulatory “immune-suppressing” T cell during sepsis. Overall, Treg percentages decreased tremendously in the spleen at day 7 following CLP. However, the relative amount of Tregs in the total CD4^+^ T helper cell subset significantly increased in the spleen and MLN which is in accordance with previously reported data, both in rodents and in human subjects in the later stages of immunoparalysis [27, 56–59]. In this matter, lymphocyte apoptosis is considered to play an important role in the setting of immunoparalysis [28, 29]. In this experimental setup, we could not find a significant difference between the percentage of TUNEL positivity between control and septic animals. It appears that in this mild animal model of sepsis, apoptosis of lymphocytes in the colon does not contribute to a considerable degree to the septic state. One could deduce however from the flow cytometry data from splenic tissue that a prolonged septic state does correlate with a decrease in the absolute percentage of CD3+ cells. Apoptosis of other immune cells, such as neutrophils, may also play a role in the prognosis of sepsis and/or containment of the septic process, but we have not studied this due to the limited amount of tissue samples.

The definition of what exactly constitutes “the” dendritic cell, or “the” regulatory T cell, is still a matter of debate [60, 61]. Monocytes, macrophages, and dendritic cells, three categories of cells pertaining to the phagocytic system, are classified based on their functional and phenotypical characteristics [60, 62]. In this manuscript, we determined MHCII^+^CD11c^+^ to be dendritic cells based on the literature and previously performed experiments in our lab. CD11c is, however, also commonly expressed on the cell surface of other phagocytic cells such as macrophages, and MHCII is furthermore present on the cell surface of group 3 innate lymphoid cells [54]. As such, we cannot state with certainty that the DC that we identified is actually not a macrophage. Ideally, inclusion of the cell surface marker CD64 or IgG1 receptor could be suggested for future experiments, as conventional DCs do not express this marker [60, 61]. There is currently still a need for a “pan-dendritic cell” expression marker, such as the Flt3ligand as has been proposed by Schraml et al. and others [61, 63, 64]; one might preferentially refer to a subset of cells based on their expression pattern of membranes (and intracellular) markers (e.g., MHCII^+^CD11c^+^ cell), which tells us the probable function and plausible properties of the cell subset, instead of simply insisting on naming the population (e.g., in this case “dendritic cell”).

Gut DCs, more specifically *lamina propria* DCs, were then typically subdivided into three major categories based upon their expression of the cell surface markers: CD103^+^CD11b^+^ DCs, CD103^−^CD11b^+^ DCs, and CD103^+^CD11b^−^DCs. Besides its potency to induce Tregs, it is accepted that the subset of CD103-expressing DCs is particularly capable of, upon encountering an antigen, migration towards the draining MLN and induction of the priming of naïve T cells towards CCR9 and *α*4*β*7 integrin-expressing T cells, which will subsequently differentiate into gut-homing T cells [60, 65, 66]. The proportion of CD11b^+^CD103^−^ DCs and CD11b^−^CD103^+^ DCs in our control animals is for the greater part in accordance with those reported by Bekiaris et al*.,* although we measured a slightly larger subset of CD11b^+^CD103^−^ DCs in the lamina propria of ileum and colon tissues, with the final subset considered to be nonmigratory and derived from monocytes [60, 67–69]. The number of CD103^+^ DCs in the spleens of sham animals was limited as well [70]. In septic animals, we observed a significant rise of the relative contribution of CD103-expressing DCs to the overall number of DCs in the spleen, whereas their percentages dropped significantly in the colon and ileum. This could be indicative of the migration of CD103^+^ DCs from the gut. No differences were, however, observed in the MLN, a feature one might expect considering the migratory potential of DCs during inflammation. A significant rise in the number of “nonmigratory” CD11b^+^CD103^−^ DCs was observed in the gut by day 7 coinciding with a decrease in the spleen and MLN, suggesting that a more inflammatory substrate, such as the CD11b (or integrin alpha M), mediates leukocyte adhesion and cell-mediated cytotoxicity, a feature that was confirmed in the colon and ileum by a significant rise in the percentage of NK cells. Moreover, as CD11b^+^CD103^−^ DCs are especially capable of inducing IFN-*γ* secretion from T cells, these results are in accordance with the increased concentrations of IFN-*γ* that we measured in the colonic supernatants at day 7 post-CLP [60]. Finally, the same subset of CD11b^+^ lamina propria DCs is specialized in driving Th17 responses, a feature we confirmed by detecting increased levels of IL-17 protein and mRNA in colonic supernatants and tissue, respectively [71].

As demonstrated in this manuscript, flow cytometry can be a powerful tool in the study and characterization of immune cells in the blood as well as different tissue and organs during sepsis. The use of appropriately matched FMO controls can be difficult, as we could isolate no more than approximately 2 to 5 million LPMCs from the colonic lamina propria prelevated from a single 10-week-old OF-1 mouse, which is in accordance with literature [25]. This limited the number of staining panels that can be applied in one single rodent, as current guidelines recommend to use sufficient cells per staining in order to correctly identify the so-called “rare events” [45–47].

The above findings confirm that the CLP model, when ligating 50% of the cecum's length followed by a single puncture with a 25G needle, displays a biphasic course of disease. The initial sickness “peak” was characterized by an increase in proinflammatory cytokines such as IL-6 and TNF-*α* in the serum and colon, and an influx of neutrophils into the bowel wall. The second sickness peak was characterized by the concurrent increase in CD11b^+^ DCs and release of IL-17A and IFN-*γ* in the colon, making it plausible that the release of these cytokines could be mediated directly or indirectly via the stimulation of CD4^+^ T cells by this DC subset [71]. Additional intracellular staining experiments are required in order to confirm this hypothesis.

Some features of a true anergic or CARS phase were confirmed at day 7, such as the marked drop in T cells (lymphopenia) in the spleen, the MLN, and the blood. Besides, serum levels of the anti-inflammatory cytokine TGF-*β* were increased and a trend towards increased IL-10 serum levels was noted; the latter two findings could, however, also be observed at day 2, reflecting a more simultaneous activation of the pro- and anti-inflammatory system during sepsis. Of note, it is currently acknowledged that features of both extreme phenotypes can be observed simultaneously in the average septic patient, instead of perseveringly trying to label them as being simply pertaining to one of both categories [30]. Septic animals at day 7 also displayed a significant drop in their rectal temperature, a sign that reflects profound lymphopenia in patients and that is associated with higher mortality [72]. This suggests that hypothermia could be a suitable early marker of sepsis-induced immunosuppression.

## 5. Conclusions

In this series of experiments, we provide an extensive quantitative description of the different types of gastrointestinal immune cells that play a role in sepsis-induced gastrointestinal tract alterations. Septic animals displayed an impaired gastrointestinal motility at day2 and day 7 after the induction of sepsis, making the model qualified for studying novel compounds that tackle the gastrointestinal motility changes that occur during sepsis. We confirm the gastrointestinal tract to be an important target in the battle against sepsis. Caution is, however, warranted, as different effects could be observed in this CLP model on different immune subsets and immune parameters in different organs, and at different time points. A myriad of markers should therefore always rigorously be taken into account when performing additional studies or interventions. The data observed here ought to be confirmed in human tissue samples, which may ultimately lead to the identification of new targets during sepsis.

## Figures and Tables

**Table 1:** Identification of immune cells in each panel based upon expression of cell surface markers.

Antibody	Cell population
*Panel 1—dendritic cell stain*	
MHCII^+^CD11c^+^CD11b^+^	CD11b + dendritic cells (proinflammatory) (CD11b^+^DCs)
MHCII^+^CD11c^+^CD103^+^	CD103^+^ dendritic cells (anti-inflammatory) (CD103^+^ DCs)
*Panel 2—macrophage/mast cell stain*	
F4/80^+^	Macrophages (M*φ*)
IgE^+^CD117^+^	Mast cells (MC)
*Panel 3—T cell and B cell stain*	
CD3^+^CD4^+^	T helper cells
CD3^+^CD8^+^	Cytotoxic T cells
CD19^+^	B cells
*Panel 4—regulatory T cell stain*	
CD4^+^CD25^+^Foxp3^+^	Regulatory T cells (Tregs)
*Panel 5—neutrophil/natural killer cell/monocyte stain*
CD335^+^	Natural killer cells (NKs)
Ly6C^+^	Monocytes (Mo)
Ly6G^+^	Neutrophils (NPh)

The different subsets of leukocytes that were identified during the flow cytometry experiments based upon the expression of cell surface markers and their abbreviations as used in this manuscript between round brackets.

**Table 2:** Cell blood count and white blood cell differentials.

Parameter	sham	CLP day 2	CLP day 7
Hemoglobin (g/dl)	12.88 ± 0.74	11.97 ± 0.64	2.81 ± 0.73^∗∗∗^
Erythrocytes (×10^6^/*μ*l)	8.32 ± 0.39	7.62 ± 0.42	1.78 ± 0.48^∗∗^
Thrombocytes (×10^3^/*μ*l)	1067.00 ± 64.17	517.6 ± 91.68^∗^	1894 ± 196.6^∗∗^
Leukocytes (×10^3^/*μ*l)	2.21 ± 0.12	2.30 ± 0.30	7.23 ± 1.84^∗^
Neutrophils (%)	4.20 ± 0.58	4.87 ± 0.50	2.33 ± 0.62^∗^
Lymphocytes (%)	59.12 ± 6.98	23.30 ± 3.12^∗^	22.19 ± 2.97^∗^
Monocytes (%)	30.24 ± 7.62	63.24 ± 3.42^∗^	60.79 ± 4.42^∗^
Eosinophils (%)	0.40 ± 0.11	0.22 ± 0.10	0.29 ± 0.07
Basophils (%)	1.50 ± 0.83	1.87 ± 0.86	0.44 ± 0.10
Undifferentiated/immature (%)	5.56 ± 0.91	7.56 ± 1.45	11.88 ± 1.39^∗^

Cell blood count and white blood cell differential of whole blood obtained by cardiac puncture in septic (CLP day 2 or CLP day 7) and control animals (sham); the different subsets of leukocytes are expressed as percentage of the leukocyte population. One-way ANOVA with post hoc Dunnett test or Kruskal–Wallis test with post hoc Dunn's test as appropriate. *n* = 6/group. ^∗^ *p* < 0.05, ^∗∗^ *p* < 0.01, and ^∗∗∗^ *p* < 0.001.

**Table 3:** Cytokines in the serum and colon following CLP-induced sepsis.

	Sham	CLP day 2	CLP day 7	*p* value
(a) *Serum cytokines (pg/ml)*				
IL-6	2.57 ± 0.48	243.60 ± 54.89^∗^	35.39 ± 10.97	<0.0001
IL-10	0.59 ± 0.32	2.33 ± 1.25	2.82 ± 1.82	NS
TNF-*α*	4.95 ± 0.29	63.72 ± 21.25^∗^	23.89 ± 3.61	0.008
IL-2	<L/D	0.38 ± 0.16	<L/D	/
IL-17A	0.21 ± 0.05	0.35 ± 0.71	3.57 ± 0.61^∗^	0.006
IFN-*γ*	<L/D	1.12 ± 0.44	0.89 ± 0.53	NS
TGF-*β*	152.50 ± 14.33	194.10 ± 12.18^∗^	198.10 ± 13.68^∗^	0.04
(b) *Colonic cytokines (pg/100 mg colonic tissue)*				
IL-6	103.3 ± 18.95	1904 ± 874.6	3410 ± 1429	0.1108
IL-10	0.79 ± 0.39	6.08 ± 1.47^∗^	5.42 ± 1.71^∗^	0.0163
TNF-*α*	2.53 ± 0.76	6.57 ± 1.46^∗^	12.38 ± 3.84^∗^	<0.0001
IL-2	<L/D	<L/D	<L/D	/
IL-17A	0.87 ± 0.29	0.31 ± 0.10	2.43 ± 1.05	0.06
IFN-*γ*	0.76 ± 0.34	1.53 ± 0.47	8.09 ± 4.77	0.141
(c) *mRNA colonic cytokines*				
IL-6	1.27 ± 0.45	28.32 ± 14.79	4.14 ± 1.88	0.096
IL-10	1.06 ± 0.15	9.59 ± 6.30	4.83 ± 1.66	0.317
TNF-*α*	1.00 ± 0.03	3.04 ± 0.78^∗^	10.29 ± 0.49^∗^	<0.001
IL-17A	1.19 ± 0.30	1.59 ± 0.68	19.54 ± 6.97^∗^	0.004
IFN-*γ*	1.42 ± 0.49	0.80 ± 0.15	2.73 ± 0.73^∗^	0.007
CRP	1.23 ± 0.39	8.21 ± 3.05^∗^	2.20 ± 0.45	0.025
IL-1 beta	1.11 ± 0.21	3.96 ± 0.45^∗^	6.47 ± 1.65^∗^	0.004
TLR4	1.06 ± 0.17	0.63 ± 0.29	0.56 ± 0.27	0.327
IL-1 alpha	1.14 ± 0.23	1.53 ± 0.43	2.20 ± 0.61	0.239

Cytokine levels in serum and colonic supernatants, as determined by CBA (or ELISA for TGF-*β*) for protein content and real-time RT-PCR for mRNA content in colonic tissue. One-way ANOVA followed by Dunnett post hoc testing or its nonparametric equivalent as appropriate; ^∗^ *p* < 0.05 for the Dunnett post hoc test compared to the sham group. For the PCR-results, data are expressed as relative expression (2^−ΔΔCT^ method) and the sham group (left column) was chosen as calibrator. Data are presented as mean ± SEM. *n* = 10 animals per group serum cytokines, *n* = 5–11 animals per group for colon cytokines, and *n* = 6 animals per group for the PCR. <L/D: below the limit of detection; IFN: interferon; IL: interleukin; TNF: tumor necrosis factor.

## Data Availability

The datasets used for the statistical analysis will be made available as Supplementary Materials to the readers.

## Abbreviations

%GE: Percentage of gastric emptying
°C: Degrees Celsius
*μ*g: Microgram
*μ*l: Microliter
*μ*M: Micromolar
ANOVA: Analysis of variance
CARS: Compensatory anti-inflammatory response syndrome
CBA: Cytometric bead array
cm: Centimeter
CD: Cluster of differentiation
CDS: Clinical disease score
CLP: Cecal ligation and puncture
CLPd2: CLP animals sacrificed at day 2
CLPd7: CLP animals sacrificed at day 7
DC: Dendritic cell
FACS: Fluorescence-activated cell sorting
FMO: Fluorescence minus one
Foxp3: Forkhead box p3
FSC: Forward scatter
g: Gravity
G: Gauge
GAPDH: Glyceraldehyde phosphate-3 dehydrogenase
GI: Gastrointestinal
h: Hour
IFN: Interferon
IL: Interleukin
iNOS: Inducible nitric oxide synthase
i.p.: Intraperitoneally
LGM: Lymphocyte growth medium
LPMC: Lamina propria mononuclear cells
LPS: Lipopolysaccharide
MCP-1: Monocyte chemoattractant protein-1
mg/kg: Milligram per kilogram
min: Minutes
ml: Milliliter
MLN: Mesenteric lymph nodes
mm: Millimeter
mM: Millimolar
MPO: Myeloperoxidase
PI: Propidium iodide
POI: Postoperative ileus
rpm: Rounds per minute
s.c.: Subcutaneously
SEM: Standard error of the mean
SIRS: Systemic inflammatory response syndrome
SSC: Side scatter
TGF: Tumor growth factor
TNF: Tumor necrosis factor
Treg: Regulatory T cell
U: Units.

## Supplementary Materials

Supplementary Materials File *Database Tcel and Treg panels CLP model* contains the raw data for the flow cytometric analysis of the T cell and B cell populations. File *Database Flowcytometrie nieuwe panels CLP model* contains the raw data for the flow cytometric analysis of the other cell populations.
