# Supplementary material for: Identifying Therapeutic Targets for Sepsis Research: A Characterization Study of the Inflammatory Players in the Cecal Ligation and Puncture Model
Source: Mediators Inflamm. 2018 Aug 5;2018:5130463. doi: 10.1155/2018/5130463 (PMC6098915; doi:10.1155/2018/5130463)
Supplement: Supplementary Materials — File Database Tcel and Treg panels CLP model contains the raw data for the flow cytometric analysis of the T cell and B cell populations. File Database Flowcytometrie nieuwe panels CLP model contains the raw data for the flow cytometric analysis of the other cell populations. [file 5130463.f1.pdf]

Supplementary table 1 The *clinical disease score* (CDS) as applied in our study to score signs of disease in individual mice.

Sign	***Score***
Piloerection (not present – extensive)	0-2
Conjunctivitis (not present – bilateral)	0-2
Grooming behaviour (normal – none)	0-2
Mobility (normal – reduced – immobile)	0-3
Signs of peritoneal irritation (none – tiptoeing and broad pace)	0-2
Position of the ears (erect – flat)	0-1
Stool consistency (normal – sticky or diarrhea)	0-1
Anemic appearance (absent or present)	0-1
Moribund	0-1

Total *Clinical Disease Score* (no signs of disease – 0-15 maximum)

Supplementary Table 2 Specifications of the BD Accuri ™ C6 flow cytometer

BD Accuri™ C6 flow cytometer
488 nm and 640 nm laser, 3 blue 1 red configuration
Optical filter FL1 533/30 nm (FITC, BB515)
Optical filter FL2 585/40 (PE, PE-CF594, PI)
Optical filter FL3 > 670 nm (PerCP-Cy5.5, PE-Cy7, PI)
Optical filter FL4 675/25 nm (APC, AF647)

Supplementary table 3 Immune cell populations stained with the characterization experiments. The different antibodies used for the flow cytometry experiments.

**Antibody**	**Manufacturer (reference)**	**Clone**
**Panel 1 – dendritic cell stain**		
Rat anti-mouse CD11b BB515	BD Horizon (564454)	M1/70
Hamster anti-mouse CD11c PE-CF594	BD Horizon (562454)	HL3
anti-mouse I-A/I-E (MHCII) PerCPCy5.5	Biolegend (107625)	M5/114.15.2
anti-mouse CD103 APC	Biolegend (121413)	2E7
**Panel 2 – macrophage / mast cell stain**		
Rat anti-mouse IgE FITC	eBioscience (11-5992)	23G3
Rat anti-mouse CD117 PE	eBioscience (12-1172)	ACK2

Rat anti-mouse F4/80 APC	eBioscience (17-4801)	BM8
**Panel 3 – T and B cell stain**		
Rat anti-mouse CD3 FITC	BD Pharmingen (561798)	17A2
Rat anti-mouse CD8a PE	BD Pharmingen (553032)	53-6.7
Rat anti-mouse CD4 PerCPCy5.5	BD Pharmingen (550954)	RM4-5
Rat anti-mouse CD19 APC	BD Pharmingen (561738)	1D3
**Panel 4 – regulatory T cell stain**		
Rat anti-mouse CD25 FITC	BD Pharmingen (561798)	17A2
Rat anti-mouse CD4 PerCPCy5.5	BD Pharmingen (550954)	RM4-5
Rat anti-mouse Foxp3 AF647	BD Pharmingen (560401)	MF23
**Panel 5 – neutrophil / natural killer cell / monocyte stain**		
Rat anti-mouse CD11b BB515	BD Horizon (564454)	M1/70

Rat anti-mouse CD335 PE	BD Pharmingen (560757)	29A1.4
Rat anti-mouse Ly6G PE-Cy7	BD Pharmingen (560601)	1A8
Rat anti-mouse Ly6C APC	BD Pharmingen (560595)	AL-21

Supplementary table 4 Cell surface markers used in the flow cytometric analysis of mouse leukocytes. The different cell surface markers that were applied during the flow cytometry experiments in order to characterize different subsets of leukocytes. APC: antigen presenting cell; CD: cluster of differentiation; Ig: immunoglobulin; NK: natural killer; TCR:T cell receptor. A simplified overview based upon current knowledge and adapted from Abbas *et al.*, 2013.

**Marker (CD)**	**Signifies...**	**Found on...**
CD103	Integrin alpha E	intraepithelial lymphocytes, lamina propria T cells, subset of gut mucosa and MLN dendritic cells
CD11b	Integrin alpha M (macrophage-1 antigen, complement receptor 3)	innate immune cells (monocytes, macrophages, dendritic cells, natural killer cells)
CD11c	Integrin alpha X	dendritic cells, monocytes, macrophages

CD117	Mast/stem cell growth factor receptor / proto-oncogene c-Kit	mast cells, interstitial cells of Cajal
CD19	B-lymphocyte antigen CD19	B cells
CD25	α-chain interleukin-2 receptor	activated T and B cells, regulatory T cells
CD3	complex consisting out of the γ, δand two ε-chains of the T cell-receptor	T cells
CD335	Natural cytotoxicity triggering receptor 1 (activation receptor), synonym NKp46	NK cells
CD4	co-receptor of the T cell receptor (TCR)	T helper cells, monocytes
CD8a	co-receptor of the TCR, recognition of antigens presented by APCs	Cytotoxic T cells

Foxp3	regulator in the development and differentiation of immune suppressing regulatory T cells	Regulatory T cells
F4/80	cell surface glycoprotein	murine macrophages
Ly6G	unknown; neutrophil migration (?)	neutrophils
I-A/I-E (MHCII)	major histocompatibility complex II, found on APC	dendritic cells, mononuclear phagocytes, B cells
IgE	Immunoglobulin E	basophils, mast cells
Ly6C	co-stimulatory signal for T cell activation, homing of cytotoxic T cells	monocytes

Supplementary Table 5 Changes in absolute percentage of different leukocyte populations in gastrointestinal tissues following CLP-induced sepsis

	Spleen		MLN		Ileum LPMC		Colon LPMC	
	sham → *CLPd2*	*sham →* *CLPd7*	*sham →* *CLPd2*	*sham →* *CLPd7*	*sham →* *CLPd2*	*sham →* *CLPd7*	*sham →* *CLPd2*	*sham →* *CLPd7*
**T cells**	+7.44%	-70.79%*	+4.51%	-32.33%*	-1.02%	-10.02%	-24.50%	-20.97%
**T helper cells**	-5.49%	-79.32%*	-2.35%	-40.61%*	-24.50%	-21.09%	-9.73%	-16.81%
**Cytotoxic T cells**	+11.18%	-78.88%*	+18.12%	-17.67%	-8.56%	-6.16%	-52.21%	-47.06%
**Tregs**	+46.39%[a]	-64.46%*	+43.54%	-8.16%	+35.00%	+10.00%	+20.00%	+26.67%
**B cells**	+10.56%	-71.47%*	-0.38%	+26.47%*	-8.02%	-34.77%[b]	-44.12%	-47.19%
**Dendritic cells**	-33.11%	-67.57%*	-19.20%	-4.80%	+104.76%*	+111.11%*	-9.84%	+134.43%*
**$CD103^+$ DCs**	-18.18%	-18.18%	-4.55%	+4.55%	+71.43%	-7.14%	+10.00%	-30.00%
**$CD11b^+$ DCs**	-9.09%	-86.36%*	-4.55%	-34.09%	+23.53%	+138.24%*	-38.24%	+179.41%*

**Macrophages**	+66.98%*	-18.42%	+64.17%[c]	+97.08%*	-5.00%	-67.00%*	-14.86%	-72.97%*
**Mast cells**	+93.65%	+283.33%*	+57.73%	+197.94%*	+110.53%	+172.63%	+135.24%	+64.76%
**Monocytes**	+3.07%	+2.17%	-3.64%	+3.08%	+16.16%	+60.70%	-23.77%	+91.47%*
**Natural killer cells**	-42.89%*	-19.67%	+24.81%	+447.29%*	+7.36%	+99.13%*	+59.14%	+165.59%*
**Neutrophils**	+23.49%	+97.44%*	+31.29%	+122.79%*	+25.00%	+31.63%	+239.86%*	+115.38%*

Percentage increase or decrease in the absolute number of leukocyte subset in the overall leukocyte gate (based upon FSC and SSC properties) in comparison to the sham-group. One-way ANOVA with post-hoc Dunnett or non-parametric equivalent as appropriate, with the sham-group as control. *$p < 0.05$; [a]$p = 0.086$; [b]$p = 0.067$; [c]$p = 0.075$ for the post-hoc Dunnett. N = 10-12/group for spleen and MLN, n = 6-7/group for ileum and colon LPMC.

CLPd2: cecal ligation and puncture sacrificed at day 2; CLPd7: cecal ligation and puncture sacrificed at day 7; DC: dendritic cell; LPMC: lamina propria mononuclear cells; MLN: mesenteric lymph nodes; Tregs: regulatory T cells
